# Helping Optimize Language Acquisition (HOLA) Online Parent Training Modules for Latinx Parents of Toddlers at Risk for ASD: Protocol for a Pilot Funded by the Organization for Autism Research

**DOI:** 10.2196/18004

**Published:** 2020-12-10

**Authors:** Robin Lynn Dodds

**Affiliations:** 1 California State University Los Angeles, CA United States

**Keywords:** autism spectrum disorders, cultural diversity, parent training, pivotal response treatment, health disparities, online training, autism, intervention delay, online learning, pediatrics

## Abstract

**Background:**

Culturally competent parent training in evidence-based intervention for autism spectrum disorder (ASD) can provide young Latinx children from underserved communities with early interventional support while they wait for professional services, thus reducing the impact of intervention delays. Providing parents with brief bilingual training in Pivotal Response Treatment (PRT) is a strategy that can overcome these barriers and is inexpensive to disseminate. Brief PRT training has been shown to significantly improve joint attention, expressive language, responsivity, and adaptive skills in young children with ASD. However, it is unknown whether an interactive, culturally competent online parent training in PRT is effective in a Latinx population.

**Objective:**

To this end, we will recruit 24 children (16-36 months old) at risk for ASD and their parent(s) from East and South Los Angeles and provide them with a series of 6 online learning modules in their choice of Spanish or English.

**Methods:**

This pilot study will utilize a single-group, pilot, pre-post design with follow-up assessments 6 weeks later. Linear mixed-effects model analysis will be used to explore most parent-reported and coded outcomes.

**Results:**

Brief online parent training in evidence-based treatments has the capacity to increase access to culturally competent early communication interventions for young children at risk for ASD.

**Conclusions:**

The results of this trial may have particular salience in additional underresourced communities where children have limited access to interventions prior to entering school.

**International Registered Report Identifier (IRRID):**

PRR1-10.2196/18004

## Introduction

### Background

Early access to evidence-based intervention leads to best-case, long-term outcomes for children with autism spectrum disorder (ASD) [1], with the greatest developmental gains experienced by children who begin intervention before their third birthday [2,3]. A recently published meta-analysis revealed that intervention benefits to social communication begin to diminish before a child turns 4 years, highlighting the importance of ASD-focused early intervention services in the developmental period [4]. Delay in the receipt of ASD-focused intervention has been associated with later autism severity, placement in segregated learning environments, and lower scores on tests of student achievement [5].

Parents from underserved communities often have less knowledge about ASD and are less likely to recognize ASD symptoms [6,7]. Therefore, their children are less likely to receive an early diagnosis of ASD. This marks a missed opportunity for intervention during the critical developmental period. Additionally, underserved families may find interventions that require a significant time commitment or cause their child to react emotionally untenable due to cultural mismatch [8].

Targeted outreach to early childhood centers and coordination with early intervention systems and health care providers, paired with dissemination of culturally competent, parent-mediated intervention models are crucial to reduce this service-need discrepancy for underserved families [9]. White, middle to upper class participants represent a significant majority in quality research studies of evidence-based practice for young children with ASD, so it is yet unclear whether established treatments are effective in nonprivileged or racially or ethnically diverse samples [10]. Virtual parent training interventions for ASD that have shown merit utilize telehealth models [11,12] that are limited by inflexible scheduling, as parents must be available to attend at predetermined times, require hard-copy hand-outs, and are limited to English-proficient participants. To make parent-mediated interventions more accessible to diverse samples of families, it has been suggested that clinicians make training materials less complicated, allow for flexibility in how the program is delivered, and teach families how to practice the intervention within their preexisting routines [13,14].

### Research With Latinx Families of Children With ASD

According to recent qualitative research on the implementation of evidence-based interventions for ASD in Latinx families, parents frequently have limited knowledge regarding ASD and require additional information. However, they are ultimately eager to participate in teaching their child and focused on helping them improve their functioning [15]. Latinx mothers of young children with ASD may also have a heightened sense of guilt regarding their child’s diagnosis, possibly believing it a punishment for something they have done in the past [16], making the delivery of accurate, culturally informed Spanish language information and intervention even more crucial [17]. In addition to these barriers, the climate of anti-immigrant sentiment and media coverage of the Immigration and Customs Enforcement detention centers and family separations may keep Latinx families who need services for their young children from seeking help.

Providing Latinx parents with brief online training in Pivotal Response Treatment (PRT) strategies in their language of preference can help overcome barriers and reduce disparities. Brief PRT training has been shown to significantly improve joint attention, expressive language, responsivity, and adaptive skills in young children with ASD [18,19]. And a recently published report on a pilot study of an online PRT parent training reported significant improvements in parent fidelity of implementation and social communication in a diverse sample of young children [20]. However, brief online PRT training has not been tested for effectiveness in a bilingual Latinx sample.

Latinx families have rapidly grown to be among the highest users of smartphone technology [21], and recent eHealth studies have shown preliminary success in using mobile technology to increase knowledge regarding diagnosis and improve health-related behaviors [22]. To this end, we are in the process of developing a smartphone-optimized, interactive, bilingual, culturally competent brief online PRT training curriculum titled “Helping Optimize Language Acquisition” (HOLA) with feedback from an advisory committee comprised of a bilingual parent of a young child with ASD, a PRT expert with over 20 years of experience in parent-mediated PRT coaching, and a bilingual Early Intervention (EI) program administrator. The HOLA curriculum is based on the information, lessons, and examples included in “Using Pivotal Response Treatment to Teach First Words to Children with Autism” [23] and “Pivotal Response Treatment: Using Motivation as a Pivotal Response” [24] by Koegel, as well as information from “Parents Taking Action,” a culturally informed bilingual training program for parents of children with ASD or developmental disabilities [25]. HOLA will be delivered to parents in 6 brief (20-40 minute) weekly modules in English or Spanish (Textbox 1).

**Topics addressed in each of the 6 modules in the Helping Optimize Language Acquisition (HOLA) program.**

Module 1

- Child Development
- Autism Spectrum Disorder

Module 2

- Child Choice
  Getting Started
  Natural Environments
  Favorite Things!
  What is HOLA?

Module 3

- Providing Natural Rewards
  What are Natural Rewards?
  Rewarding Good Trying
  Easy Tasks

Module 4

- Providing Clear Opportunities
  Getting Your Child’s Attention
  Providing a Clear Opportunity
  Responding Right Away

Module 5

- Putting it All Together
  Reviewing the Strategies
  Teaching in Daily Routines
  Celebrating Your Child

Module 6

- Planning for the Future
  Troubleshooting
  Task Variety
  Family Balance
  Taking Care of Yourself

**Figure 1:**
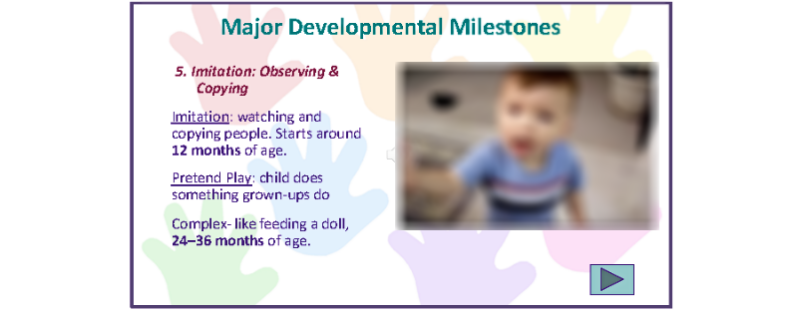
Example video enhanced page from an HOLA module.

**Figure 2:**
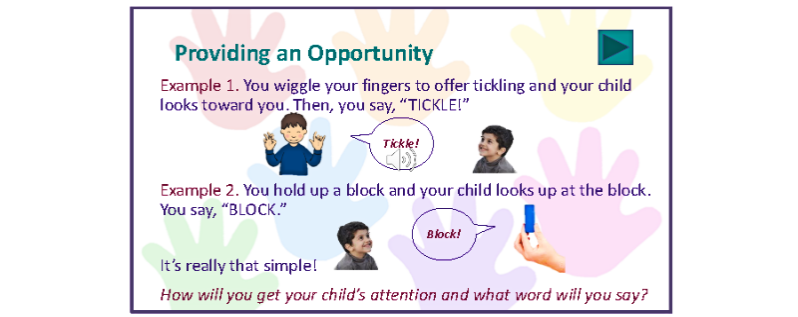
Example interactive page from an HOLA module.

The HOLA modules will differ from other virtual parent trainings in that they will be accessible online at any time of day, they can be viewed multiple times, and all needed materials will be integrated into the training modules. The HOLA training will make use of adult learning principles and use multiple modalities including presentations, visual representations, gamification, and video examples to teach the content, and text will be simplified and supported by visuals to allow for participants of varying levels of literacy (Figures 1 and 2). These training strategies have been shown to improve learning outcomes when compared to traditional presentation methods such as narrative slideshows for a variety of learners [26].

## Methods

### Objectives and Study Design

This pilot study will utilize a single-group, pilot, pre-post design with follow-up assessments 6 weeks later. All assessments described in the subsequent paragraphs are available in English and Spanish. It is hypothesized that the HOLA parent training will improve parent knowledge of ASD and child development, increase fidelity of implementation of the strategies taught, and improve child social communication. Additionally, we predict that the parent will experience gains in empowerment and reductions in parenting stress, and the children will have reductions in challenging behavior and display improved social skills. Finally, we predict that there will be no difference in participant outcomes between Spanish and English language trainings.

To test the HOLA intervention, we will recruit 24 Latinx toddler-parent dyads (16-36 months old) with elevated risk for ASD from the East and South Los Angeles regions to measure the effects of the HOLA intervention on parent knowledge of child development, parent PRT fidelity of implementation, and child social communication. We will also measure parent satisfaction with the intervention and explore the impact of HOLA on parent stress, family empowerment, and child behavior. Additionally, because participants will choose whether they would prefer to receive the intervention in Spanish or English, we will assess whether language has an impact on effectiveness of and satisfaction with HOLA.

All screening and assessment activities as well as data entry and monitoring for this study will take place in the Parent Education and Support (PEAS) Lab of author RD in the Charter College of Education at California State University, Los Angeles (Cal State LA). The PEAS Lab is located in the same building as the Robert L. Douglass Speech and Language Clinic, Center for Multicultural Education, C. Lamar Mayer Learning Center, and Center for Excellence in Early Intervention and Low Incidence Disabilities. This dedicated research space includes an assessment room; a waiting area for families; access to family-friendly, American Disabilities Act–compliant bathroom facilities; and 3 additional computers/workstations for data entry, transcription, coding, statistical analysis, and data storage. Specifically, the subject testing room consists of a dedicated 8-foot by 8-foot room containing a chair for caregivers, toddler-sized furniture, child-friendly furnishings, and keyed storage for toys and assessment materials.

Centro de Niños y Padres (located next door to the PEAS Lab) is a family-focused program providing early intervention services to children from birth to age 3 years, who have or are at risk for a developmental disability. The Centro de Niños y Padres program serves families in the neighboring culturally diverse communities through developmentally appropriate practices. The program serves children who have been identified as having developmental disabilities and children who are at risk, including those with complex and intensive medical needs. Infants and toddlers without disabilities whose parents are interested in a family-centered early learning environment dedicated to diversity and celebration of the family role in development and learning are also welcome to participate in the program. Although many of the children served by Centro de Niños y Padres have a range of abilities, many of the children they serve exhibit delays in social communication, making it an appropriate agency for recruitment of families who may qualify for participation in the proposed study.

### Recruitment and Consent Procedures

The Cal State LA Institutional Review Board (IRB) will approve all recruitment and consent procedures prior to enrollment begins. All study staff will complete IRB research training through Cal State LA. Recruitment will take place in 2 ways: through referrals from entities that serve young children and through targeted outreach. The primary recruitment site for this project will be Centro de Niños y Padres early intervention program. Additional sites identified for referrals include Federally Qualified Community Health Centers, Family Resource Centers, and the Diagnostic Clinics, all programs that serve children with disabilities from low-resource communities in the Los Angeles area. Presentations on ASD and free screening events will be scheduled for parents and staff at childcare and Early Head Start locations in proximity to the university as well as pediatric practices to facilitate recruitment. Parents of children identified as having elevated concern for ASD will be informed about potential participation in our research study. The referring organization will proceed, as appropriate, with standard referrals (EI, Child Find), regardless of interest in participating in our research study. The partnering organization will provide referral contact information (study flyer) to the project staff for all families that indicate interest and consent to be contacted by the project staff.

Once a telephone or email inquiry or referral from a partnering organization is received, project staff will call the family to explain the purpose and procedures of the study. If the participant indicates interest in participation, project staff schedule an assessment appointment with the family. The potential participant will complete a screening assessment consent form, which will allow the assessor (principal investigator [PI] for English, bilingual research assistant for Spanish) to administer the Modified Checklist for Autism in Toddlers, Revised, with Follow-Up (MCHAT), which is a parent-report screening tool to assess autism risk [27]. If the child scores below 3 on the MCHAT or does not meet all eligibility requirements, the parent will be compensated for the visit (US $25) and receive an assessment report, and all study materials will be destroyed. The assessor will continue with baseline assessments if the child and family meet all eligibility criteria: child is between 16 and 36 months of age, is minimally verbal or pre-verbal, scores 3 or above on the MCHAT, and has no other physical or sensory disabilities.

Before starting baseline assessments, the assessor will review the informed consent form in the appropriate language with the participant, and any questions will be answered. The participant will be told that this is a research study, that his or her participation will not positively or negatively affect the provision of services to the family, and that he or she is free to decline to answer any questions or to cease participation at any point. The confidentiality of the information will be assured. The participant will be told that no identifying information will be retained on any of the data collection forms and that a unique and nonidentifying ID code number will be assigned in lieu of his or her name. The participant will be asked to sign 2 copies of the informed consent (1 for the participant to keep, 1 for the project files), after which baseline assessment will begin. Participants will then receive 6 weekly links to the online

modules in their language of preference as outlined in the following sections.

### Considerations for Families From Low Income and Diverse Communities

Some families, especially from communities of color, may have had negative experiences that result in a lack of trust in the research community. Further, they may perceive research participation as lacking benefit to them and their child. Bonevski and associates [28] outlined strategies to improve trust and participation in low-resource and culturally diverse communities in a systematic review. They highlight a need for increased flexibility in scheduling, the provision of monetary or tangible incentives, reduced travel to the research site, and capitalizing on connections within the community of interest [28]. These recommendations have been incorporated in the development of this study, so that the assessor will schedule appointments with the parent to accommodate their schedule and preferences, participants will receive compensation for their time after each assessment visit, and community-based EI providers and medical practices that accept Medicaid reimbursement will be the main referral points for the study.

Baseline assessments will be conducted with all children and parents prior to participation in the study. The purpose of the screening and baseline assessments is to confirm ASD risk and assess parent, child, and family characteristics prior to their receipt of intervention. In addition, posttest (6 weeks after the start of intervention) and follow-up (6 weeks postintervention) assessments will be conducted to assess changes in parent and child characteristics over time. Parents will receive US $25 for their time for completing screening or baseline, posttest, and follow-up assessments (US $75 total). All time point assessments will involve travel to Cal State LA.

### Enrollment and Participation

Over 1 year, 24 parent/child dyads will participate in the study. The parents will receive 6 weekly emails or text messages, each with an active link to access an online training module. All electronic data will be stored in a password-protected university server, and only IRB-authorized personnel will be granted password access.

### Screening Measures

For autism risk, the MCHAT [27] is a parent-report autism screening tool for children 16 to 30 months of age. It will be collected pre-intervention. Children who score above 3 will qualify to participate in the study and will be referred for a diagnostic evaluation.

### Control Measures

#### Demographic Questionnaire

The demographic questionnaire will be created by investigators. The questionnaire will include questions regarding parent age, education, income, employment, English language proficiency, health status, child age, and gender. It will be collected pre-intervention.

#### Service Use Questionnaire

The service use questionnaire will be created by the PI. Parents will be asked to indicate which services the child is currently receiving, including the number of hours received weekly for each service. Services may include applied behavior analysis therapy, occupational therapy, physiotherapy, speech therapy, recreational or social activities, or education-based services (EI, preschool). Services used and hours of services will be counted for a total score and will be examined individually. This will be collected pre-intervention, postintervention, and at follow-up.

#### Recruitment Site

I will control for recruitment site, as participants recruited from different sites may have had very different experiences related to their child’s development and represent distinct demographic groups. This will be collected pre-intervention.

### Primary Outcome Measures

#### Social Communication

Social communication will be coded from a recorded play session between the parent and child with a standard set of toys using frequency counts to identify the presence of the communicative behavior (vocalizations, eye contact, or positive affect) over a 5-minute video-recorded play session. The overall percentage of intervals in which the children used any type of social communication will be calculated. This will be collected pre-intervention, postintervention, and at follow-up.

#### Parent Fidelity of Implementation

Parent Fidelity of Implementation will be scored using a continuous 1-minute interval recording system over a 10-minute video segment of a recorded play session between the parent and child with a standard set of toys. Parents will be scored for the correct use of each of 5 PRT principles (presenting clear opportunities, child choice, immediate contingent responses, natural reinforcers and reinforcing verbal attempts, and correct verbal responses), and an overall percentage will be calculated. This will be collected pre-intervention, postintervention, and at follow-up.

#### Satisfaction

Parents will complete a survey rating their satisfaction with their training on a 5-point Likert scale. Items will include, but are not limited to, accessibility, helpfulness, convenience, and thoroughness of training program and materials. Space for parent suggestions will also be provided. This will be collected postintervention.

#### Child Development Knowledge

To assess child development knowledge, a researcher-created measure will be used that was developed to assess parent knowledge of typical and atypical development. This will be collected pre-intervention, postintervention, and at follow-up.

### Secondary Outcome Measures

#### Overall Language

The Preschool Language Scales Fifth Edition [29] is a norm-referenced, play-based, comprehensive developmental language assessment for children from birth to age 7 years. This will be collected pre-intervention, postintervention, and at follow-up.

#### Expressive Language

The MacArthur Bates Communicative Development Inventories Words and Gestures Form [30] is a standardized, parent-report form that tracks young children's language and communication skills including words the child understands, words the child uses, and gestures tried or completed. This will be collected pre-intervention, postintervention, and at follow-up.

#### Parent Stress

The Parenting Stress Index-Short Form [31] is a self-report screening tool that helps providers and families identify the sources and different types of stress that come with parenting. The 36 items are divided into 3 domains that combine to form a Total Stress scale: Parental Distress, Parent-Child Dysfunctional Interaction, and Difficult Child. This will be collected pre-intervention, postintervention, and at follow-up.

#### Empowerment

The Family Empowerment Scale [32] is a 24-item self-report measure that evaluates levels of empowerment experienced by parents or other caregivers of children with emotional or behavioral challenges. This will be collected pre-intervention, postintervention, and at follow-up.

#### Challenging Behavior

The Child Behavior Checklist 1.5-5 [33] obtains caregivers’ self-report ratings of 99 problem items. Items are scored on the following scales: Emotionally Reactive, Anxious/Depressed, Somatic Complaints, Withdrawn, Attention Problems, Aggressive Behavior, and Sleep Problems. The assessment also includes open-ended questions to obtain additional qualitative information about child problems and strengths. This will be collected pre-intervention, postintervention, and at follow-up.

#### Social Skills

The Ages & Stages Questionnaire-Social Emotional is a parent-report measure focused on social and emotional development in young children. The Ages & Stages Questionnaire-Social Emotional provides insight into important developmental areas, such as self-regulation, communication, autonomy, compliance, adaptive functioning, affect, and interaction with people. This will be collected pre-intervention, postintervention, and at follow-up.

### Sample Size Determination

A recently published report on a monolingual, brief, online, PRT pilot reported significant improvement in parent fidelity of implementation of PRT strategies and measures of child social communication (vocalizations, eye contact, and positive affect) after 5 weeks, with large effect sizes of 1.80 and 1.12, respectively [20], similar to findings from earlier studies. Therefore, given a conservative estimated effect size of 0.8 for our primary outcomes between baseline and postintervention timepoints, a sample size of 13 is needed to have 80% power to detect a significant difference in primary outcomes between these timepoints given an α of .05. Given our goal of enrolling 24 participant dyads and assuming normal distributions, we can estimate 97% power to detect a significant difference in primary outcomes between baseline and postintervention.

### Quantitative Analysis

Linear mixed-effects model analysis will be used to explore most parent-reported and coded outcomes because (1) it allows for individual differences in parent responses over time to be included in the model as a random effect so that the variance in responses is partitioned into a time-within-parent component and a between-parent component, (2) using maximum likelihood estimation in a linear mixed-effects model allows for the use of all available data to evaluate the parameter values and is currently considered the state-of-the-art method for handling missing data, and (3) linear mixed-effects models perform well with small samples. The linear mixed-effects models will evaluate time as the main fixed effect using discrete time variables (ie, 2 dummy variables with baseline as the “referent”). Separate regression models will be run for each outcome. Additional variables to be included in each regression model will include total score of other services used, recruitment site, and only demographic variables significantly related to the outcome (given the small sample size).

Cohen d effect sizes will be calculated between time periods and reported for all analyses of interest as a measure of the magnitude of any changes in outcome measures. Mixed models will also be utilized to test if changes from baseline on outcome variables differ by parent language preference for training (Spanish or English) over time. HOLA acceptability will be determined by the average parent rating on the Parent Satisfaction measure. If the average ratings for parent satisfaction items are 3.5 or higher, the training may remain as written. Items with parent satisfaction ratings lower than 3.5 will be explored during Spanish and English focus groups, and individual items will be refined according to feedback. Focus group transcripts will be coded and analyzed thematically to inform continued program improvement.

### Qualitative Analysis

Focus groups comprised of a subsample of Spanish-speaking and English-speaking parents who have completed the HOLA intervention and follow-up activities will be gathered either on campus or by Zoom. The focus groups will last between 1 and 2 hours, and topics related to intervention satisfaction, alignment with cultural beliefs, and socially valid outcomes will be discussed using a flexible protocol that includes open-ended questions and follow-up probes. The facilitator (bilingual graduate research assistant/PI) will engage all participants and allow additional topics to emerge as necessary, while being sure to discuss all protocol questions. The focus groups will be audio-recorded and transcribed verbatim. Analysis will proceed in 3 stages following a modified grounded theory procedure [34]. Initial codes will be written in the margins, actively summarizing but remaining close to the original text. Next, focused coding will proceed where the most frequent and significant codes will be used to synthesize and organize the data. Finally, codes will be sorted into theoretical categories, and relationships among codes and theoretical categories will be hypothesized [34]. Data from focus groups will inform HOLA intervention improvements and may identify areas of participant growth not measured by quantitative assessments.

## Results

In a recent commentary on the state of current ASD research, Kasari and Smith [35] recommended additional research be conducted with diverse samples of children with ASD who are minimally verbal to determine whether evidence-based interventions in common use are effective in underrepresented populations. As much of the research on evidence-based interventions for ASD has utilized single-subject designs and homogeneous samples, a trial of a brief bilingual PRT intervention in a Latinx sample will provide the field with valuable insights. The HOLA intervention is simple, cost-effective, and scalable because it can be delivered to parents directly by computer modules accessible by wireless technology and can be easily embedded into existing family routines. As wireless access has expanded, nearly 80% of Latinos in the United States own a smartphone, with those under 30 years old using their cellphone for most of their online activities [21], and cell phone–based interventions have been popular and effective in addressing health disparities in this and other underserved groups [36]. While HOLA is not intended to replace more intensive interventions for ASD, it may provide parents of at-risk or newly diagnosed children with important knowledge about child development and autism symptoms and bridge the gap in access to services for underserved Latinx children.

HOLA modules will teach parents to follow their toddler’s lead and respond to and reinforce their child’s communicative attempts in order to increase child motivation and engagement and improve social, cognitive, and language development. Because most children in underserved communities are unlikely to receive early intervention services for ASD, a missed opportunity to develop crucial social communication, the development of culturally informed bilingual parent-mediated brief PRT interventions like HOLA could bring quality, flexible, evidence-based interventions to toddlers at risk for ASD while awaiting diagnosis. The HOLA intervention will also increase parenting efficacy by improving a parent’s ability to engage with their child through play and daily routines such as mealtime and bath time and may have additional benefits related to decreased child challenging behavior.

To my knowledge, there are no published trials of ASD-focused, parent-mediated interventions designed for Latinx families. As Latinx children are consistently underdiagnosed and underserved by the ASD community, it is crucial to provide parents with knowledge and strategies to assist their child in developing language. The HOLA intervention will require large-scale randomized controlled trials to provide robust evidence of effectiveness in Latinx and additional underserved populations, utilizing fewer parent-report and more observational measures.

The results of this pilot study will be used in the preparation of an application to the National Institute of Deafness and Communication Disorders Early Career Award or to the Institute of Education Sciences Research Training Programs in Special Education Early Career Award for a multiyear randomized controlled trial of the HOLA intervention. Future plans for the HOLA bilingual online training include free public access to all training modules and multilingual peer support pages, as well as links to high-quality resources for families and service providers by way of a university website paired with outreach to local and national disability and autism-focused organizations and childcare providers to inform stakeholders of HOLA's availability. Translation of modules into additional languages including Chinese, Tagalog, Korean, Armenian, Vietnamese, Farsi, Japanese, and Russian should be considered in order to provide access to families who speak the top 10 languages in Los Angeles County.

## Discussion

HOLA will help Latinx parents create rich learning environments for their children in Spanish or English, by teaching them how to embed language development opportunities into everyday routines and to improve the reciprocity in their relationship with their child. Our program focus will educate parents about child development and the signs of ASD, thereby improving parents’ ability to communicate concerns to appropriate service providers and gain access to diagnostic referrals and quality services. HOLA will also empower parents to better support their child’s academic progress through culturally informed advocacy and provide them with information on the importance of self-care when raising a child with learning differences in order to maintain emotional balance and improve family quality of life. Taken together, these outcomes may reduce stigma and depression experienced by Latinx mothers of children with disabilities, reduce the age of ASD diagnosis, and reduce service disparities for Latinx children.

## Appendix

Multimedia Appendix 1 Peer-review report.

## Abbreviations

ASD: autism spectrum disorder
Cal State LA: California State University, Los Angeles
EI: Early Intervention
IRB: Institutional Review Board
MCHAT: Modified Checklist for Autism in Toddlers, Revised, with Follow-Up
PEAS: Parent Education and Support
PI: principal investigator
PRT: Pivotal Response Treatment
