# Supplementary material for: Helping Optimize Language Acquisition (HOLA) Online Parent Training Modules for Latinx Parents of Toddlers at Risk for ASD: Protocol for a Pilot Funded by the Organization for Autism Research
Source: JMIR Res Protoc. 2020 Dec 10;9(12):e18004. doi: 10.2196/18004 (PMC7759438; doi:10.2196/18004)
Supplement: Multimedia Appendix 1 [file resprot_v9i12e18004_app1.pdf]

OAR Research Grant

Michael Maloney <mmaloney@researchautism.org>
Thu 11/14/2019 12:28 PM

To: Dodds, Robin L <rdodds@calstatela.edu>
Cc: Kimberly Ha <kha@researchautism.org>; Kristen Essex <kessex@researchautism.org>

1 attachments (32 KB)
OAR Applied Budget Form.xlsx;

Dr. Dodds,

Let me add my congratulations to those you received this week from OAR Board member, Anthony Ferrera, on being selected as an OAR research grantee.

In the course of reviewing your proposal, members of the review panel offered the following comments relative to your proposed study. Additionally, I have reviewed the budget and other administrative requirements. Please review the combined comments below and respond accordingly by Thursday, November 21. An e-mail response will suffice.

Review Comments.

Help Optimize Language Acquisition (HOLA): On-line Parent Training Modules for Latino Parents of Children at Risk for ASD.

- Brief On-line (smart phone optimized) Parent Training in PRT for Latino Community
- They have direct observation measures of parents and kids
- ***Is it possible for the researchers to include additional language measures normed in Spanish?***

Administrative Comments:

a. Submit proof of IRB Approval
b. Color printing and binding of manuals is considered an administrative cost. The updated administrative cost is $5669.40, or 14% of the total budget. Please submit a revised budget using the attached budget spreadsheet template.

Dr. Dodds, please respond to the question posed and administrative comments via e-mail. After we receive your reply and proof of IRB approval, we will send the grant agreement to your sponsored research office. Once we receive the signed grant agreement back, we will schedule the first installment of the grant.

Should you have any questions, please contact me, (703) 243-9727; mmaloney@researchautism.org. Good luck in your research efforts.

Sincerely,

Mike

Michael V. Maloney

Executive Director
Organization for Autism Research
2111 Wilson Boulevard, Suite 401
Arlington, VA 22201
(P) (703) 243-9727 (F) (703) 243-9751
mmaloney@researchautism.org
www.researchautism.org
www.operationautism.org

This message is intended for the sole use of the individual and entity to which it is addressed and may contain information that is privileged, confidential and exempt from disclosure under applicable law. If you are not the intended addressee, nor authorized to receive for the intended addressee, you are hereby notified that you may not use, copy, disclose or distribute to anyone the message or any information contained in the message. If you have received this message in error, please immediately advise the sender by reply email and delete the message. Thank you.
